# Microbial production of the plant flavanone hesperetin from caffeic acid

**DOI:** 10.1186/s13104-023-06620-8

**Published:** 2023-11-18

**Authors:** Erik K. R. Hanko, João Correia, Caio S. Souza, Alison Green, Jakub Chromy, Ruth Stoney, Cunyu Yan, Eriko Takano, Diana Lousa, Cláudio M. Soares, Rainer Breitling

**Affiliations:** 1 Manchester Institute of Biotechnology, Faculty of Science and Engineering, University of Manchester, 131 Princess Street, Manchester, M1 7DN UK; 2 Instituto de Tecnologia Química e Biológica António Xavier, Universidade Nova de Lisboa, Av. da República, 2780-157 Oeiras, Portugal

**Keywords:** Hesperetin, Flavonoid, Biosynthesis, *O*-methyltransferase, Enzyme engineering, Homoeriodictyol, Combinatorial library

## Abstract

**Objective:**

Hesperetin is an important *O*-methylated flavonoid produced by citrus fruits and of potential pharmaceutical relevance. The microbial biosynthesis of hesperetin could be a viable alternative to plant extraction, as plant extracts often yield complex mixtures of different flavonoids making it challenging to isolate pure compounds. In this study, hesperetin was produced from caffeic acid in the microbial host *Escherichia coli*. We combined a previously optimised pathway for the biosynthesis of the intermediate flavanone eriodictyol with a combinatorial library of plasmids expressing three candidate flavonoid *O*-methyltransferases. Moreover, we endeavoured to improve the position specificity of CCoAOMT7, a flavonoid *O*-methyltransferase from *Arabidopsis thaliana* that has been demonstrated to *O*-methylate eriodictyol in both the *para*- and *meta*-position, thus leading to a mixture of hesperetin and homoeriodictyol.

**Results:**

The best performing flavonoid *O*-methyltransferase in our screen was found to be CCoAOMT7, which could produce up to 14.6 mg/L hesperetin and 3.8 mg/L homoeriodictyol from 3 mM caffeic acid in *E. coli* 5-alpha. Using a platform for enzyme engineering that scans the mutational space of selected key positions, predicting their structures using homology modelling and inferring their potential catalytic improvement using docking simulations, we were able to identify a CCoAOMT7 mutant with a two-fold higher position specificity for hesperetin. The mutant’s catalytic activity, however, was considerably diminished. Our findings suggest that hesperetin can be created from central carbon metabolism in *E. coli* following the introduction of a caffeic acid biosynthesis pathway.

**Supplementary Information:**

The online version contains supplementary material available at 10.1186/s13104-023-06620-8.

## Introduction

Flavonoids are a structurally diverse class of polyphenolic natural products that have a wide range of health-promoting effects [1–3]. Hesperetin is a prominent example of the flavanone subclass that has been extensively studied for its anti-inflammatory, cardioprotective, anticancer, and antimicrobial properties [4]. It is primarily found in citrus fruits in its glycosylated form, hesperidin [5]; extraction and enzymatic deglycosylation can be applied to yield hesperetin [6].

As an alternative to plant-based extraction, hesperetin can be biosynthesised by microbial cell factories. *Escherichia coli* has recently been harnessed to produce hesperetin using two different methods. The first strategy utilised the natural pathway by which plants synthesise hesperetin. It involves the cytochrome P450-dependent hydroxylation of naringenin in the 3′-position to form eriodictyol, followed by an *O*-methylation in the 4′-position that is catalysed by a flavonoid *O*-methyltransferase (OMT) [7]. The second strategy avoids expression of a flavonoid OMT. Instead, it was based on a promiscuous flavonoid biosynthesis pathway that utilises isoferulic acid, a phenylpropanoic acid with a methoxy group at the *para*-position, as a substrate [8]. However, despite efforts to rationally engineer chalcone synthase, the enzyme that synthesises the flavonoid backbone, its performance against the *para*-*O*-methylated substrate remains poor [9].

In this study, we demonstrate the biosynthesis of hesperetin from caffeic acid in the microbial host *E. coli*. Using a chassis that can generate the flavanone eriodictyol from caffeic acid, we evaluate a combinatorial library of plasmids containing three flavonoid *O*-methyltransferases for their ability to convert eriodictyol into hesperetin. Moreover, we demonstrate that the flavonoid *O*-methyltransferase CCoAOMT7, which can *O*-methylate eriodictyol in both the *para*- or *meta*-position, resulting in hesperetin or homoeriodictyol, respectively, can be engineered to improve its position specificity towards hesperetin. This was achieved using an enzyme engineering platform that seeks to predict the most favourable mutants for improving this specificity.

### Methods

#### Base strains and media

NEB (New England Biolabs) 5-alpha competent *E. coli* was used for cloning, plasmid propagation, and flavonoid biosynthesis. *E. coli* strains were routinely propagated in Luria–Bertani medium [10]. For production of flavonoids, cells were grown in phosphate-buffered Terrific Broth (TBP, Formedium) supplemented with 0.4% (v/v) glycerol. When required, antibiotics were added to the media at the following concentrations: 34 µg/mL chloramphenicol, 50 µg/mL kanamycin.

#### Cloning and transformation

Plasmid DNA was purified using the QIAprep Spin Miniprep Kit (Qiagen). DNA was amplified by PCR in 50 µL reactions using the Q5 High-Fidelity 2 × Master Mix from NEB. The Zymoclean Gel DNA Recovery Kit (Zymo Research) was employed to extract gel-purified linearised DNA. NEBuilder HiFi DNA Assembly Master Mix, restriction enzymes, and T4 DNA ligase were purchased from NEB. All PCR-, HiFi-, digestion-, and ligation reactions were set up following the manufacturer’s instructions. Chemically competent *E. coli* were prepared and transformed by heat shock [10].

#### Enzyme selection

To ensure that an extensive range of flavonoid *O*-methyltransferases (OMTs) were considered, the enzyme selection tool Selenzyme was used to supplement literature searches [11]. This tool requires that the query reaction is represented as a simplified molecular-input line-entry (SMILE), assembled from compound canonical SMILES sourced from PubChem [12]. This query was run using default parameters, and CCoAOMT7 was among the top results. The other enzymes, SOMT-2 and CrOMT, were selected from the literature.

#### Plasmid construction

Oligonucleotide primers were synthesised by IDT and are listed in Additional file 1: Table S1. Gene parts were designed using PartsGenie with the RBS translation initiation rates set to 20,000 [13]. Flavonoid OMTs were optimised for *E. coli* codon usage and parts were synthesised by Twist Bioscience. The sequences of the synthesised DNA fragments can be found in Additional file 1: Table S2. Combinatorial library plasmids were generated by digestion of synthesised DNA fragments using *Eco*RI/*Bam*HI restriction enzymes followed by ligation into selected BglBrick vectors (Addgene) that had been linearised using the same enzymes [10, 14]. Plasmids carrying the mutant CCoAOMT7s were constructed by HiFi DNA Assembly (NEB), and a detailed description of how each of them was built can be found in the Supplementary Methods. Correct assembly was validated by Sanger sequencing (Eurofins Genomics). All plasmids used and generated in this study are listed in Additional file 1: Table S3.

#### Biosynthesis of flavonoids

To test the individual strains for flavonoids production, single colonies of freshly transformed cells were used to inoculate 1 mL of TBP supplemented with 0.4% glycerol, 50 µg/mL kanamycin with or without 34 µg/mL chloramphenicol. Seed cultures were grown in 96-deepwell plates sealed with breathable plate seals overnight at 30℃ and 75% humidity with orbital shaking at 850 rpm. To set up the main cultures, seed cultures were diluted to an OD_600nm_ of 0.02 into 1 mL of fresh medium and returned to the shaker-incubator. At an OD_600nm_ of 1.0–1.5 cultures were supplemented with isopropyl β-d-1-thiogalactopyranoside (IPTG) and caffeic acid to final concentrations of 100 µM and 3 mM, respectively. When required, cerulenin was added to a final concentration of 20 µg/mL. Cultures were returned to the shaker-incubator and samples were taken after 24 h.

#### Quantification of target compounds

Caffeic acid, eriodictyol, hesperetin, and homoeriodictyol were quantified by LC–MS/MS analysis. To prepare the extract samples, cultures were quenched with an equal volume of 100% methanol, mixed by vortexing, and clarified by centrifugation at 16,000 *g* for 10 min. Cell-free supernatants were diluted 100- or 1000-fold using methanol/water (10:90 v/v). LC–MS/MS analysis was performed as reported previously using a Waters ACQUITY ultra-performance liquid chromatography (UPLC) H-Class System coupled to a Xevo TQ-S triple-quadrupole mass spectrometer (Waters) equipped with an electrospray ionisation (ESI) source [15]. All four compounds were monitored in ESI^−^ mode. The LC method is described in the Supplementary Methods. MS method parameters for each individual compound, including precursor ion mass, product ion mass, cone voltage, and collision energy are listed in Additional file 1: Table S4.

#### Protein modelling and docking

To predict the structure of CCoAOMT7, a homology-based model of the enzyme was created with Modeller [16], using the X-ray structure of *Medicago sativa* caffeoyl coenzyme A 3-*O*-methyltransferase (PDB ID: 1SUI) [17], which has a protein sequence identity of 59%, and the model of the target protein generated using AlphaFold2 [18, 19]. To identify enzyme mutants with improved position specificity towards hesperetin, the differential binding of both products was used as a proxy for differential catalytic efficiency. Docking calculations in the enzyme’s active site for hesperetin and homoeriodictyol were performed using AutoDock Vina [20]. The box size was set to 13 × 16 × 23 Å^3^ and the exhaustiveness to 200. To ensure that both reacting atoms are at a catalytically relevant distance the results were filtered to only keep conformations that display a maximum distance of 4.1 Å between the *O*-methyl carbon of the ligand and the sulphur atom of the product *S*-adenosyl homocysteine (SAH). Additionally, a minimum distance of 5.0 Å was kept between the adjacent hydroxyl oxygen atom of the ligand and the sulphur atom of SAH, and a maximum distance of 9.7 Å was maintained between the carbon in the ligand that is most distant from the reactive part and a residue at the opposite site. The mutation space was limited to residues within the active site. Subsequently, all possible combinations of single and double mutants were generated and evaluated. The above steps were performed automatically by the in-house gene discovery and enzyme engineering (GDEE) platform. Mutant candidates were selected by manual curation based on the conformation and orientation of both ligands in the active site and the estimation of protein–ligand interactions, according to the free binding energies provided by AutoDock Vina. Conformations and orientations with more catalytic relevance and with the lowest binding free energies were preferred.

## Results and discussion

### Biosynthesis of hesperetin from caffeic acid

**Fig. 1:**
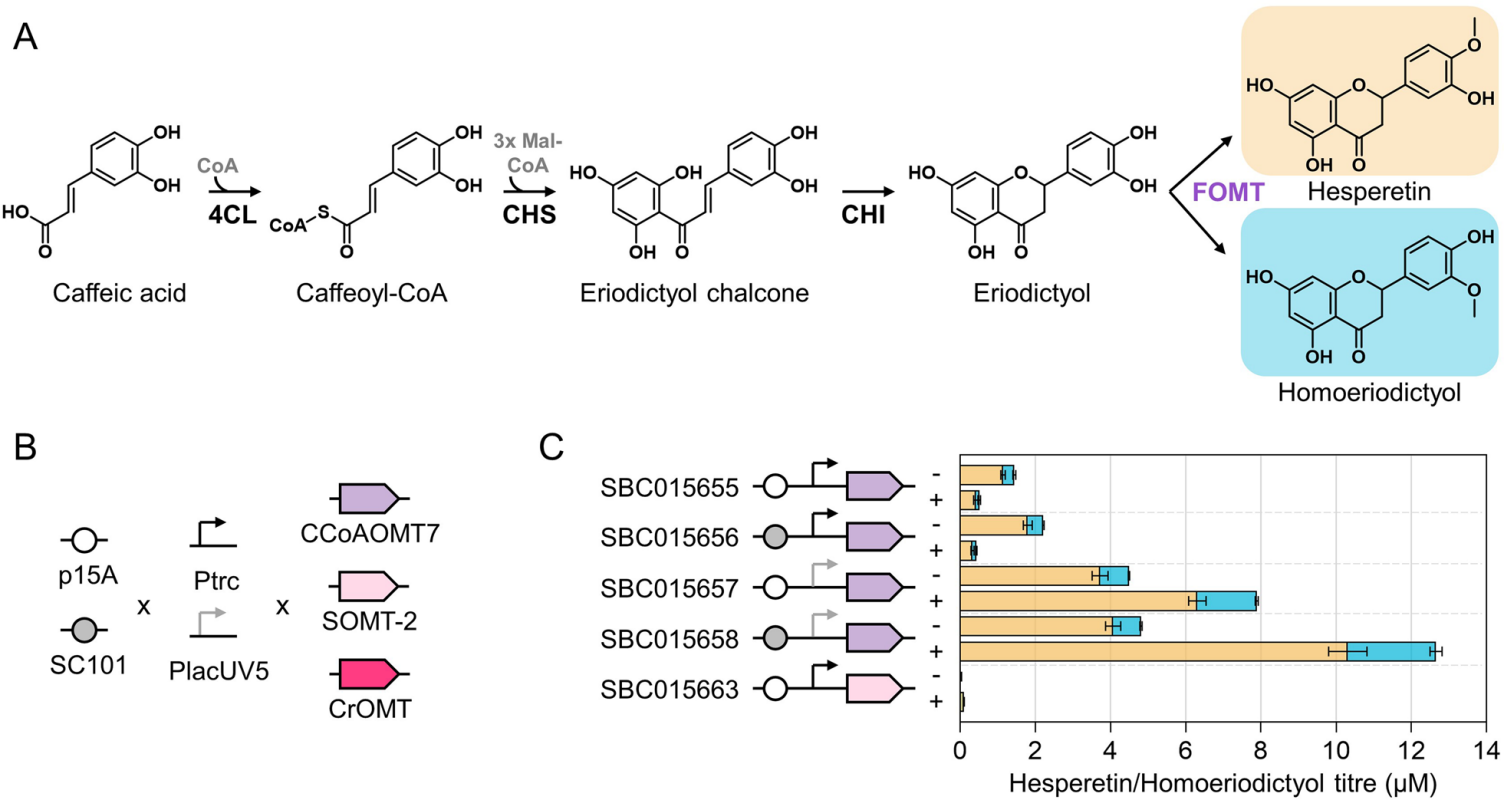
Microbial biosynthesis of hesperetin by whole-cell bioconversion of caffeic acid. **A** Hesperetin biosynthesis pathway. Enzyme abbreviations: *4CL* (4-coumarate-CoA ligase), *CHS* (chalcone synthase), *CHI* (chalcone isomerase), *FOMT* (flavonoid *O*-methyltransferase). *O*-methylation of eriodictyol in the 4′-position yields hesperetin, whereas *O*-methylation in the 3′-position yields homoeriodictyol. **B** Construction of a combinatorial library of three flavonoid OMTs (FOMTs) using two types of origins of replication (p15A and SC101) and two varying-strength promoters (stronger promoter, P_*trc*_; weaker promoter, P_*lacUV5*_). **C** Strains producing hesperetin (orange) and homoeriodictyol (blue). To produce hesperetin and homoeriodictyol from caffeic acid, *E. coli* 5-alpha was cotransformed with the eriodictyol biosynthesis pathway (encoded by plasmid SBC006845) and the combinatorial library of plasmids carrying the flavonoid OMTs as illustrated in **B**. Error bars represent standard deviations of three biological replicates. Strains were grown in the absence (−) and presence (+) of IPTG

Microbial production of hesperetin has previously been achieved by bioconversion of either naringenin or isoferulic acid [7, 8]. In this study, we aimed to synthesise hesperetin by whole-cell bioconversion of caffeic acid. First, a previously developed, optimised pathway was used to convert caffeic acid into eriodictyol [21]. It is composed of three enzymes: 4-coumarate-CoA ligase (4CL) from *Glycine max*, chalcone synthase (CHS), and chalcone isomerase (CHI) both from *Arabidopsis thaliana* (Fig. 1A). Subsequently, an *S*-adenosyl-l-methionine (SAM)-dependent OMT catalyses the 4′-*O*-methylation of eriodictyol to form hesperetin. We selected three flavonoid OMT enzyme candidates to be evaluated based on curation of the top enzyme hits from Selenzyme [11] and the available literature: CCoAOMT7 from *Arabidopsis thaliana*, SOMT-2 from *Glycine max*, and CrOMT from *Catharanthus roseus*. All enzymes have previously been shown to be capable of methylating eriodictyol or other structurally related flavonoids in the 4′-*O*-position [7, 22, 23].

To optimise expression of the selected flavonoid OMT enzymes, each gene was cloned into four distinct plasmid backbones. As a result, expression levels were controlled by plasmid copy number and promoter strength (Fig. 1B) [14]. Cotransformation of the plasmid encoding the eriodictyol pathway (SBC006845 [21]) and the combinatorial library of plasmids carrying the flavonoid OMTs should enable production of hesperetin from caffeic acid. The resulting *E. coli* strains were grown in rich medium, and metabolites were quantified 24 h after supplementation with 3 mM caffeic acid. Hesperetin was detectable in five out of the twelve strains (Fig. 1C). Of the three selected flavonoid OMTs only CCoAOMT7 yielded hesperetin at titres above 0.1 µM (Fig. 1C). The best performing strain, carrying SBC015658, produced 10.3 µM hesperetin. The plasmid backbone of SBC015658 contains the weaker promoter and the lower copy replication origin of the four plasmid backbones under investigation [14], suggesting that decreasing CCoAOMT7 expression levels reduced the metabolic burden on the cells leading to higher hesperetin titres. Moreover, the strain that only carried the eriodictyol pathway plasmid produced 29.5 µM eriodictyol, suggesting that the 10.3 µM hesperetin production represents a ~ 30% conversion rate (Additional file 1: Fig. S1).

In addition to the primary target compound, hesperetin, the four strains expressing CCoAOMT7 also produced homoeriodictyol. This compound has the methoxy group in the 3′-position as opposed to the 4′-position (Fig. 1A). As previously reported for in vitro biotransformation studies using CCoAOMT7 [22], hesperetin was favoured over homoeriodictyol across all four strains at a consistent 80:20 ratio. In an effort to improve the production of hesperetin, we repeated the experiment with the top-performing strain (SBC015658) in the presence of the fatty acid biosynthesis inhibitor cerulenin. Supplementation with cerulenin increases the pool of malonyl-CoA that is available to chalcone synthase (Fig. 1A) and has been shown to boost eriodictyol titres [21]. The addition of cerulenin and IPTG increased the product titres by more than 4 times, yielding 48.4 µM hesperetin and 12.7 µM homoeriodictyol (14.6 mg/L and 3.8 mg/L, respectively).

To our knowledge, this is the first instance of hesperetin being produced by bacteria from caffeic acid, which can be generated from tyrosine in two enzymatic steps [21, 24]. This suggests that hesperetin production from central carbon metabolism can be possible with further integration of a caffeic acid biosynthesis pathway. Moreover, the addition of cerulenin—which increases precursor supply—increased hesperetin titres, indicating that the current bottleneck is the production of eriodictyol, rather than its methylation. Cui and colleagues adopted a similar strategy, using isoferulic acid, a *para*-*O*-methylated phenylpropanoic acid, as the substrate for the enzymatic cascade composed of 4CL, CHS, and CHI rather than transferring a methyl group to the flavonoid as the final step [8]. However, the CHS’s poor acceptance of the activated isoferulic acid substrate remains a bottleneck of this approach [8, 9]. Furthermore, the poor performance of CrOMT matches the observations made by Liu and colleagues, who ultimately selected OMT from *Mentha* × *piperita* for the two-step biotransformation of naringenin to hesperetin [7].

### Development of CCoAOMT7 mutants with altered position specificity

**Fig. 2:**
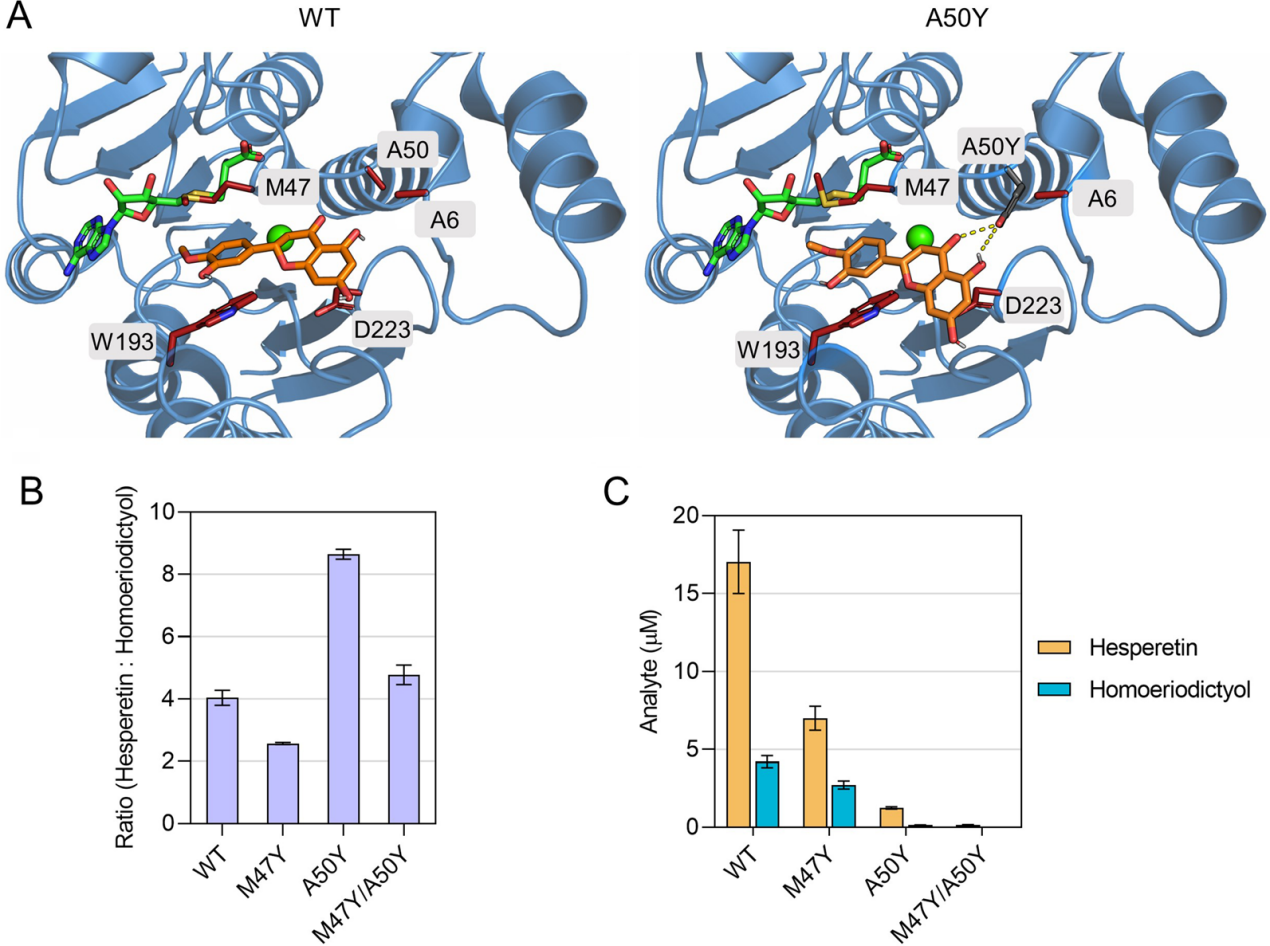
Engineering the position specificity of CCoAOMT7. **A** Homology-based model of CCoAOMT7 illustrating the interaction of hesperetin with the active site of the wild type (WT) enzyme and the A50Y mutant. Hesperetin carbon atoms are shown in orange, and active site residue sidechains are depicted as red sticks. The protein fold is represented in blue. The carbon atoms of the cofactor SAH and the calcium ion are illustrated in green. In the mutant, the carbon atoms of the Tyr50 residue are highlighted in grey, and the hydrogen bonds are indicated by dashed lines. Active site residues are labelled. **B** Ratios of hesperetin to homoeriodictyol in the three mutants and the WT CCoAOMT7. **C** Hesperetin and homoeriodictyol titres in the three mutants and the WT CCoAOMT7. Error bars represent standard deviations of three biological replicates. Strains were grown in the presence of IPTG

The ability of CCoAOMT7 to *O*-methylate eriodictyol in the *para*-position has previously been attributed to a single amino acid residue [22]. The introduction of G46Y completely reversed the enzyme’s position specificity—homoeriodictyol was preferred over hesperetin at a ratio of 80:20 [22]. Since the bioconversion of eriodictyol into hesperetin was of interest to us, we sought to engineer CCoAOMT7 to improve its position specificity towards the *para*-*O*-methylated product. As the protein structure of CCoAOMT7 was unavailable, caffeoyl coenzyme A 3-*O*-methyltransferase from *Medicago sativa* and the model of the target protein generated using AlphaFold2 [18, 19] were used as templates to create a homology-based model of the enzyme. The resulting CCoAOMT7 structure model was employed for in silico docking simulations utilising hesperetin and homoeriodictyol as ligands. Consequently, differential catalytic efficiencies were inferred from predicted product binding energies. Two single mutants were identified that exhibit higher stabilities for hesperetin in comparison to homoeriodictyol, and where the binding energies for hesperetin are lower than in the wild type enzyme (Additional file 1: Table S5). In the A50Y mutant, the hydroxyl group of the Tyr50 residue establishes two additional hydrogen bonds with the hesperetin carbonyl group at C4 and the hydroxyl group at C5 (Fig. 2A), while it establishes only one hydrogen bond with the homoeriodictyol hydroxyl group at C5 (Additional file 1: Fig. S2). The second mutant, CCoAOMT7^M47Y^, forms an additional hydrogen bond between the hydroxyl group of the Tyr47 residue and the hydroxyl group of hesperetin at C3′ (Additional file 1: Fig. S3). Both mutations were introduced into CCoAOMT7, both separately and combined, and production titres of hesperetin and homoeriodictyol were quantified. The ratio of the two products changed statistically significantly in all three mutants. While mutant CCoAOMT7^A50Y^ exhibits a more than twofold increase in position specificity toward hesperetin, in mutant CCoAOMT7^M47Y^ the ratio of hesperetin to homoeriodictyol is shifted to 72:28 (Fig. 2B). This ratio is only slightly altered to 82:18 in the double mutant. Product titres considerably decreased in all mutants, ranging from a 2.4-fold reduction in hesperetin titres in CCoAOMT7^M47Y^ to a decrease of more than 100-fold in the double mutant (Fig. 2C). In summary, these findings show that the flavonoid OMT CCoAOMT7 can be engineered to improve its position specificity towards hesperetin, albeit at the expense of catalytic efficiency and, thus, product titre.

## Limitations

In this study, we have shown that it is possible to produce hesperetin from caffeic acid using microorganisms and engineer the CCoAOMT7 enzyme to increase its position specificity. However, to further improve the product titres and enzyme specificity, additional experimentation would be required. Hesperetin titres could be increased through the optimisation of the chassis, pathway, and culture conditions. For example, the eriodictyol biosynthesis pathway and CCoAOMT7 could be combined onto a single plasmid and alternative growth media could be tested. In the long run, a combination of the hesperetin production pathway with a caffeic acid producing engineered chassis would be of particular interest. While the modelling was able to shift the product ratio of the enzyme, the decreased catalytic efficiency of the mutants is a problem, showing the fine equilibrium between many different factors and the large approximations introduced, due to the high-throughput nature of the methodology used. More complex and computationally more demanding approaches, such as the use of quantum mechanical methods, could potentially make a valuable contribution to this end.

### Supplementary Information

**Additional file 1: Table S1.** Oligonucleotide primers used in this study. **Table S2.** Sequences of the synthesised DNA fragments. Gene coding sequences are shown in uppercase letters. *Eco*RI and *Bam*HI restriction enzyme recognition sites are underlined. **Table S3.** Plasmids used and generated in this study. **Table S4.** MS method parameters for caffeic acid, eriodictyol, hesperetin, and homoeriodictyol. **Table S5.** Binding energies between the ligand flavanones and the wild type and mutant CCoAOMT7. **Figure S1.** Eriodictyol titres (µM) in strains carrying the eriodictyol biosynthesis pathway (SBC006845) in combination with the plasmids encoding the flavonoid *O*-methyltransferases (SBC015655–SBC015666) or the control plasmid pBbS5c-rfp, which expresses *rfp* instead of an OMT gene. Strains were grown in the absence and presence of IPTG. Error bars represent standard deviations of biological triplicates. **Figure S2.** Homology-based model of CCoAOMT7 illustrating the interaction of homoeriodictyol with the active site of the wild type (WT) enzyme and the A50Y mutant. Homoeriodictyol carbon atoms are shown in pale brown, and active site residue sidechains are depicted as red sticks. The protein fold is represented in blue. The carbon atoms of the cofactor SAH and the calcium ion are illustrated in green. In the mutant, the carbon atoms of the Tyr50 residue are highlighted in grey, and the hydrogen bonds are indicated by dashed lines. **Figure S3.** Homology-based model of CCoAOMT7M47Y illustrating the interaction of hesperetin and homoeriodictyol with the active site of the enzyme. Hesperetin and homoeriodictyol carbon atoms are shown in orange and pale brown, respectively, and active site residue sidechains are depicted as red sticks. The protein fold is represented in blue. The carbon atoms of the cofactor SAH and the calcium ion are illustrated in green. The carbon atoms of the Tyr47 residue are highlighted in grey, and the hydrogen bonds are indicated by dashed lines.

## Data Availability

All data generated or analysed during this study are included in this published article and its supplementary information files.

## Abbreviations

4CL: 4-Coumarate-CoA ligase
CHI: Chalcone isomerase
CHS: Chalcone synthase
DNA: Deoxyribonucleic acid
ESI: Electrospray ionisation
IPTG: β-d-1-thiogalactopyranoside
LC–MS/MS: Liquid chromatography tandem mass spectrometry
OD_600nm_: Absorbance at 600 nm
OMT: *O*-Methyltransferase
PCR: Polymerase chain reaction
SAH: *S*-Adenosyl homocysteine
SAM: *S*-Adenosyl-l-methionine
SMILE: Simplified molecular-input line-entry
TBP: Phosphate-buffered terrific broth
Tyr: Tyrosine
UPLC: Ultra-performance liquid chromatography
WT: Wild type
