# Supplementary material for: Microbial production of the plant flavanone hesperetin from caffeic acid
Source: BMC Res Notes. 2023 Nov 18;16:343. doi: 10.1186/s13104-023-06620-8 (PMC10656822; doi:10.1186/s13104-023-06620-8)
Supplement: Supplementary file 1 — Additional file 1: Table S1. Oligonucleotide primers used in this study. Table S2. Sequences of the synthesised DNA fragments. Gene coding sequences are shown in uppercase letters. EcoRI and BamHI restriction enzyme recognition sites are underlined. Table S3. Plasmids used and generated in this study. Table S4. MS method parameters for caffeic acid, eriodictyol, hesperetin, and homoeriodictyol. Table S5. Binding energies between the ligand flavanones and the wild type and mutant CCoAOMT7. Figure S1. Eriodictyol titres (µM) in strains carrying the eriodictyol biosynthesis pathway (SBC006845) in combination with the plasmids encoding the flavonoid O-methyltransferases (SBC015655–SBC015666) or the control plasmid pBbS5c-rfp, which expresses rfp instead of an OMT gene. Strains were grown in the absence and presence of IPTG. Error bars represent standard deviations of biological triplicates. Figure S2. Homology-based model of CCoAOMT7 illustrating the interaction of homoeriodictyol with the active site of the wild type (WT) enzyme and the A50Y mutant. Homoeriodictyol carbon atoms are shown in pale brown, and active site residue sidechains are depicted as red sticks. The protein fold is represented in blue. The carbon atoms of the cofactor SAH and the calcium ion are illustrated in green. In the mutant, the carbon atoms of the Tyr50 residue are highlighted in grey, and the hydrogen bonds are indicated by dashed lines. Figure S3. Homology-based model of CCoAOMT7M47Y illustrating the interaction of hesperetin and homoeriodictyol with the active site of the enzyme. Hesperetin and homoeriodictyol carbon atoms are shown in orange and pale brown, respectively, and active site residue sidechains are depicted as red sticks. The protein fold is represented in blue. The carbon atoms of the cofactor SAH and the calcium ion are illustrated in green. The carbon atoms of the Tyr47 residue are highlighted in grey, and the hydrogen bonds are indicated by dashed lines. [file 13104_2023_6620_MOESM1_ESM.docx]

**Supporting Information**

**Microbial production of the plant flavanone hesperetin from caffeic acid**

Erik K. R. Hanko^1^, João Correia^2^, Caio S. Souza^2^, Alison Green^1^, Jakub Chromy^1^, Ruth Stoney^1^, Cunyu Yan^1^, Eriko Takano^1^, Diana Lousa^2^, Cláudio M. Soares^2^, Rainer Breitling^1^*

^1^Manchester Institute of Biotechnology, Faculty of Science and Engineering, University of Manchester, 131 Princess Street, Manchester M1 7DN, United Kingdom

^2^Instituto de Tecnologia Química e Biológica António Xavier, Universidade Nova de Lisboa, Av. da República, 2780-157 Oeiras, Portugal

*Author to whom correspondence should be addressed; Email: rainer.breitling@manchester.ac.uk

# Supplementary methods

## Plasmid construction

**SBC015955** was constructed by HiFi DNA Assembly. Oligonucleotide primers EH214_f and EH215_r, EH216_f and EH217_r were used to amplify CCoAOMT7, which was split in two parts to introduce the M47Y mutation, from SBC015658. The PCR products were combined with *Eco*RI/*Bam*HI-digested pBbS5c-rfp.

**SBC015956** was constructed by HiFi DNA Assembly. Oligonucleotide primers EH214_f and EH218_r, EH219_f and EH217_r were used to amplify CCoAOMT7, which was split in two parts to introduce the A50Y mutation, from SBC015658. The PCR products were combined with *Eco*RI/*Bam*HI-digested pBbS5c-rfp.

**SBC015957** was constructed by HiFi DNA Assembly. Oligonucleotide primers EH214_f and EH215_r, EH220_f and EH217_r were used to amplify CCoAOMT7, which was split in two parts to introduce the M47Y/A50Y mutations, from SBC015658. The PCR products were combined with *Eco*RI/*Bam*HI-digested pBbS5c-rfp.

## LC-MS/MS analysis

For the separation of target compounds by liquid chromatography a Waters Acquity BEH C18 column (50 mm x 2.1 mm, 1.7 μm) was used. The column was operated at 45°C. The separation was achieved using a flow rate of 0.6 mL/min and a binary mobile phase consisting of A (H_2_O, 0.1% formic acid) and B (MeOH, 0.1% formic acid). The gradient elution program was: 0–1.5 min, 60–5% A; 1.5–1.9 min, hold at 5% A; 1.9–2.0 min, 5–60% A; 2.0–3.0 min, hold at 60% A. All samples were kept at 10°C throughout the analysis and the inject volume was 1 μL. Peak areas were integrated using MassLynx v 4.1 (Waters) software. Metabolite concentrations were quantified using calibration curves generated from running standards of known concentrations which were prepared the same as the samples.

# Supplementary Tables

Table S1. Oligonucleotide primers used in this study.

| Primer | Sequence (5′ to 3′) |
| --- | --- |
| EH214_f | agcggataacaatttcagaattcaaaag |
| EH215_r | ggtcgcataaccggcttgcggatggtt |
| EH216_f | ccggttatgcgaccgcaccggatgcg |
| EH217_r | agatccttactcgagtttggatcc |
| EH218_r | atccggataggtcgccataccggcttg |
| EH219_f | cgacctatccggatgcgggtcagctg |
| EH220_f | cggttatgcgacctatccggatgcgggtcagctg |

Table S2. Sequences of the synthesised DNA fragments. Gene coding sequences are shown in uppercase letters. EcoRI and BamHI restriction enzyme recognition sites are underlined.

| *O*-methyltransferase | Sequence |
| --- | --- |
| CCoAOMT7 | gaattcaaaagatctgagtcttgtagaacgtcgtcgccagcttgagataagctacaataggtttttattatacccgaagaagtaaagaggatctgaaggagtaccacATGGCAAAAGATGAGGCGAAAGGCTTGCTGAAAAGCGAGGAACTGTATAAGTATATTTTGGAAACCAGTGTGTACCCGCGTGAACCTGAGGTGCTCCGCGAGTTGCGCAATATCACTCACAACCATCCGCAAGCCGGTATGGCGACCGCACCGGATGCGGGTCAGCTGATGGGAATGCTGCTGAACCTGGTAAATGCCCGAAAGACGATTGAAGTTGGTGTGTTCACTGGTTACTCGTTGTTACTCACAGCATTGACCCTGCCGGAGGATGGCAAAGTTATTGCAATTGATATGAACAGGGACAGCTATGAAATCGGTTTGCCCGTTATCAAGAAGGCTGGTGTGGAACATAAAATTGACTTTAAGGAATCTGAAGCTCTGCCGGCGCTGGACGAACTGCTGAATAACAAAGTAAATGAGGGCGGCTTCGATTTTGCCTTTGTGGATGCCGATAAACTGAATTACTGGAATTATCACGAACGCCTGATCCGCTTAATCAAAGTTGGTGGGATCATTGTATACGACAATACGCTTTGGGGCGGCTCAGTCGCTGAACCCGATTCATCCACTCCAGAGTGGCGCATTGAGGTTAAGAAAGCAACCTTGGAATTAAACAAGAAGCTGTCAGCTGATCAGCGTGTGCAGATTAGCCAGGCAGCCCTTGGTGACGGCATCACCATTTGTCGTCGTCTGTACTAAttgtagactcggatccaaactcgag |
| CrOMT | gaattcaaaagatctgagtcttgtagcccgcttcttgaaaaagtcaccaccattaggccttagctacttcctacacttttcagcatgacaacttgtggggaggtaaatagcATGGATCTTCAGACTGCCGAATTTCGCGAAGCGCAGGCCAAAATCTGGTCGCAGGCATTCTCTTTCGCTAATTGCGCCGCCCTTAAATGTGCGGTCAAATTGGGTATTGCCGATGCAATTGATAACCACGACAAAAAGGCGTTAACGCTGTCGGAACTGACCGAAGAATTGAGCATCAAACCTAGTAAATCCCCATTCCTCCAGCGCTTAATGCGCCAGTTGGTGAATGCCGGCTTTTTCACGGAAGCCAAACAGCTGCGTGATGATAACAAGGATGGACGCACCACTACCGCGTACGCGCTGACCCCGGTGTCACGCCTCCTGCTGAAGAATGAACAGTGGAACCTACGCGGCATAGTATTAACAATGCTTGACCCAGCGGAGCTTAAAGCATGGAGTGTATTAAACGATTGGTTCAAAAACGATGATCCGACAGCATTCCAGACCGCCCACGAGAAAAATTATTGGGATTATACCGCTGAGAATACCCAGCACTGTCAGATTTTTGAAGACGCCATGGCCAACGATTCTGTGCTGGTGTCGAAATTACTGGTCACCGAATATAAGTTCCTCTTTGAAGGGCTGACCTCACTAATTGACCTGGGCGGCTCCACTGGAACCATTGCAAAAGCCCTGGCGAAGTCGTTTCCGAACTTAAAATGTACCGTGTTTGACTTGCCGCACGTGGTTGCCAATCTGGAGAGCACCAAGAATCTGGAATTTGTTGGCGGTGATATGTTTGAAAAACTGCCGCCGTCTAACGCCATTCTCTTGAAATGGATTCTTCATGACTGGAACGATGAAGACTGTGTTAAAATCCTTAAAAATTGTAAGAAAGCGATCCAGGAGAAAGGTAACGGTGGTAAGGTGATTATTATTGACACCGTTGTATATAGCCAAAAGAACGAAAAAGAACTGGTCGATCTGCAAATTTCTATGGATATGGCGATGGTAATTAATTTTGCCGCGAAAGAACGCACAGAAGAAGAATGGGAACATCTAATACGCGAGGCGGGGTTTAGTGGCCATAAAATTTTTCCGATGTACGATTTCCGTTCAATTATAGAAGTGTACCCTTAAttgtagactcggatccaaactcgag |
| SOMT-2 | gaattcaaaagatctgagtcttgtactcgacatggccttgcaggtggcgaccggagagtgtggcgaggtggccaacaccctcgatagaggttttacttggaggtaccATGGCGTCACCACTTAACAACGGTCGTAAAGCAAGTGAAATATTCCAGGGCCAAGCCCTGCTGTACAAGCATCTGCTCGGGTTTATTGATAGTAAATGCTTGAAGTGGATGGTGGAGCTGGATATTCCAGACATTATCCACTCACATTCGCATGGCCAACCGATCACCTTCTCGGAGCTAGTGTCGATTCTGCAGGTACCGCCTACCAAAACTCGTCAAGTGCAATCACTAATGCGTTACCTGGCGCATAACGGTTTCTTCGAAATTGTGCGCATTCACGATAATATTGAAGCCTATGCCTTGACGGCGGCCTCAGAACTGCTGGTGAAAAGCTCAGAGCTATCGCTGGCGCCAATGGTTGAATACTTTCTCGAACCCAACTGCCAGGGCGCGTGGAATCAATTAAAACGTTGGGTGCATGAAGAGGACCTCACGGTTTTTGGAGTTAGTCTGGGAACCCCATTTTGGGATTTCATTAACAAAGACCCGGCTTACAACAAATCCTTTAACGAGGCCATGGCCTGTGACAGCCAGATGCTGAACCTAGCCTTTCGCGATTGCAATTGGGTCTTTGAAGGCCTGGAAAGCATTGTCGATGTTGGGGGCGGTACCGGTATTACCGCGAAAATTATCTGTGAGGCGTTTCCTAAGCTCAAATGCATGGTTCTCGAAAGACCCAATGTTGTTGAAAATCTGAGCGGTAGCAATAACTTGACGTTTGTTGGTGGCGACATGTTTAAATGCATTCCCAAAGCGGATGCAGTACTGCTGAAACTTGTCCTTCACAACTGGAATGATAACGATTGTATGAAAATCTTAGAGAACTGCAAAGAGGCCATTAGTGGCGAGAGCAAAACTGGCAAAGTGGTCGTGATTGACACGGTTATCAACGAAAATAAGGATGAACGTCAAGTCACCGAACTTAAGCTGCTCATGGATGTTCACATGGCGTGCATTATTAATGGCAAAGAGCGCAAAGAAGAAGATTGGAAGAAACTTTTTATGGAGGCAGGTTTTCAGAGCTATAAAATTTCACCGTTTACGGGGTATCTTTCCCTGATTGAAATTTATCCTTGAttgtagactcggatccaaactcgag |

Table S3. Plasmids used and generated in this study.

| Plasmid | Characteristic | Reference or source |
| --- | --- | --- |
| pBbA1c-rfp | Cm^R^; p15A; P*_trc_* - *rfp* | [1] |
| pBbA5c-rfp | Cm^R^; p15A; P*_lacUV5_* - *rfp* | [1] |
| pBbS1c-rfp | Cm^R^; SC101; P*_trc_* - *rfp* | [1] |
| pBbS5c-rfp | Cm^R^; SC101; P*_lacUV5_* - *rfp* | [1] |
| SBC006845 | Kan^R^; colE1; P*_lacUV5_* - AtCHI - P*_trc_* - Gm4CL - P*_trc_* - AtCHS | [2] |
| SBC015553 | Cm^R^; colE1; P*_Tet_* - CCoAOMT7* | This study |
| SBC015555 | Cm^R^; colE1; P*_Tet_* - CrOMT* | This study |
| SBC015557 | Cm^R^; colE1; P*_Tet_* - SOMT-2* | This study |
| SBC015655 | Cm^R^; p15A; P*_trc_* - CCoAOMT7* | This study |
| SBC015656 | Cm^R^; SC101; P*_trc_* - CCoAOMT7* | This study |
| SBC015657 | Cm^R^; p15A; P*_lacUV5_* - CCoAOMT7* | This study |
| SBC015658 | Cm^R^; SC101; P*_lacUV5_* - CCoAOMT7* | This study |
| SBC015659 | Cm^R^; p15A; P*_trc_* - CrOMT* | This study |
| SBC015660 | Cm^R^; SC101; P*_trc_* - CrOMT* | This study |
| SBC015661 | Cm^R^; p15A; P*_lacUV5_* - CrOMT* | This study |
| SBC015662 | Cm^R^; SC101; P*_lacUV5_* - CrOMT* | This study |
| SBC015663 | Cm^R^; p15A; P*_trc_* - SOMT-2* | This study |
| SBC015664 | Cm^R^; SC101; P*_trc_* - SOMT-2* | This study |
| SBC015665 | Cm^R^; p15A; P*_lacUV5_* - SOMT-2* | This study |
| SBC015666 | Cm^R^; SC101; P*_lacUV5_* - SOMT-2* | This study |
| SBC015955 | Cm^R^; SC101; P*_lacUV5_* - CCoAOMT7^M47Y^* | This study |
| SBC015956 | Cm^R^; SC101; P*_lacUV5_* - CCoAOMT7^A50Y^* | This study |
| SBC015957 | Cm^R^; SC101; P*_lacUV5_* - CCoAOMT7^M47Y/A50Y^* | This study |

*Optimised for *Escherichia coli* codon usage.

Table S4. MS method parameters for caffeic acid, eriodictyol, hesperetin, and homoeriodictyol.

| Compound | Precursor ion mass | Product ion mass | Cone voltage (V) | Collision energy (eV) |
| --- | --- | --- | --- | --- |
| Caffeic acid | 179.0719 | 135.0888 | 32 | 14 |
| Eriodictyol | 287.0949 | 151.0526 | 2 | 14 |
| Hesperetin | 301.1119 | 164.0985 | 4 | 24 |
| Homoeriodictyol | 301.1757 | 151.0388 | 62 | 16 |

Table S5. Binding energies between the ligand flavanones and the wild type and mutant CCoAOMT7.

| Variant | Binding energy hesperetin (kcal/mol) | Binding energy homoeriodictyol (kcal/mol) |
| --- | --- | --- |
| Wild type | −7.4 | −8.0 |
| A50Y | −8.3 | −7.8 |
| M47Y | −8.4 | −7.9 |
| M47Y/A50Y | −6.2 | −6.1 |

# Supplementary Figures


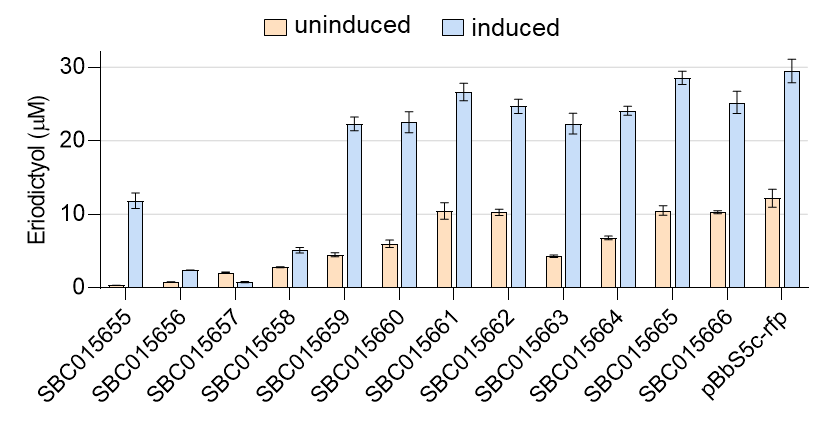


Figure S1. Eriodictyol titres (µM) in strains carrying the eriodictyol biosynthesis pathway (SBC006845) in combination with the plasmids encoding the flavonoid O-methyltransferases (SBC015655–SBC015666) or the control plasmid pBbS5c-rfp, which expresses rfp instead of an OMT gene. Strains were grown in the absence and presence of IPTG. Error bars represent standard deviations of biological triplicates.


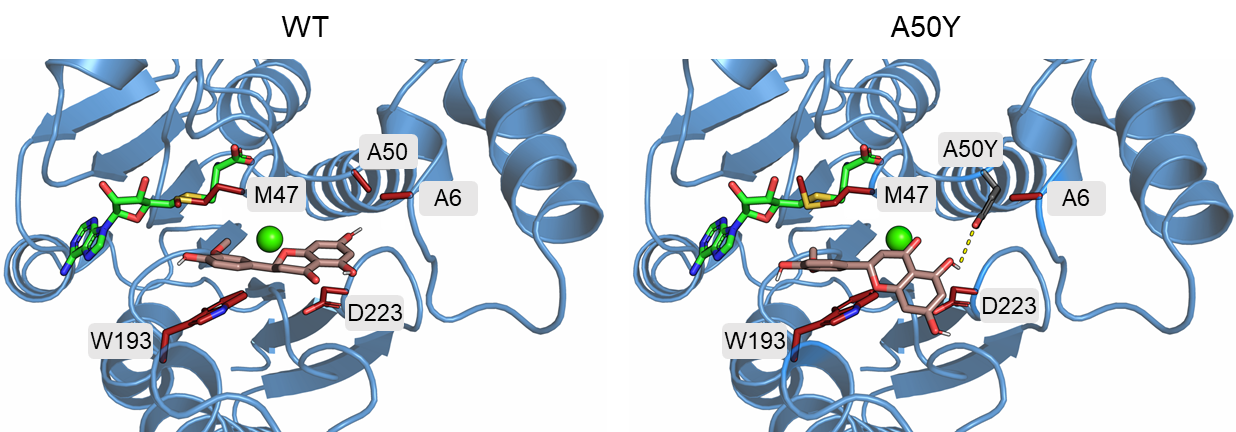


Figure S2. Homology-based model of CCoAOMT7 illustrating the interaction of homoeriodictyol with the active site of the wild type (WT) enzyme and the A50Y mutant. Homoeriodictyol carbon atoms are shown in pale brown, and active site residue sidechains are depicted as red sticks. The protein fold is represented in blue. The carbon atoms of the cofactor SAH and the calcium ion are illustrated in green. In the mutant, the carbon atoms of the Tyr50 residue are highlighted in grey, and the hydrogen bonds are indicated by dashed lines.


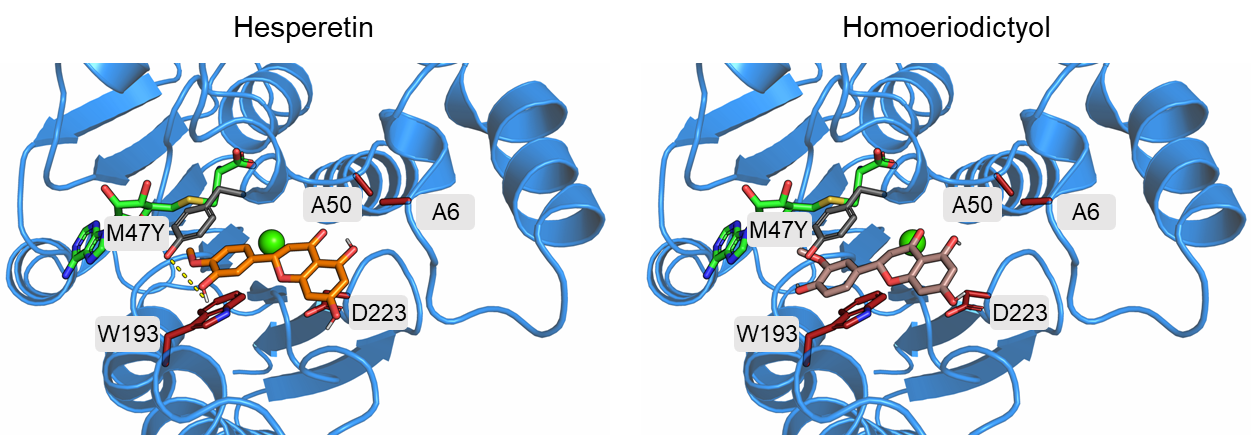


Figure S3. Homology-based model of CCoAOMT7^M47Y^ illustrating the interaction of hesperetin and homoeriodictyol with the active site of the enzyme. Hesperetin and homoeriodictyol carbon atoms are shown in orange and pale brown, respectively, and active site residue sidechains are depicted as red sticks. The protein fold is represented in blue. The carbon atoms of the cofactor SAH and the calcium ion are illustrated in green. The carbon atoms of the Tyr47 residue are highlighted in grey, and the hydrogen bonds are indicated by dashed lines.

# References

1. Lee TS, Krupa RA, Zhang F, Hajimorad M, Holtz WJ, Prasad N, et al. BglBrick vectors and datasheets: a synthetic biology platform for gene expression. J Biol Eng. 2011;5:1-14.

2. Dunstan MS, Robinson CJ, Jervis AJ, Yan C, Carbonell P, Hollywood KA, et al. Engineering *Escherichia coli* towards *de novo* production of gatekeeper (2 *S*)-flavanones: naringenin, pinocembrin, eriodictyol and homoeriodictyol. Synth Biol. 2020;5(1):ysaa012.
